# Familienbildung meets Gesundheitsförderung!? Mechanismen, Potenziale und Herausforderungen gesundheitsbezogener Förderung von Elternschaft

**DOI:** 10.1007/s11553-023-01022-x

**Published:** 2023-03-10

**Authors:** Anja Lentz-Becker, Barbara Bräutigam, Matthias Müller

**Affiliations:** Fachbereich Soziale Arbeit, Bildung und Erziehung, Hochschule Neubrandenburg University of Applied Sciences, Neubrandenburg, Deutschland

**Keywords:** Elternschaft, Salutogenese, Psychosoziale Regulationsprozesse, Ressourcen, Parenting, Salutogenesis, Regulatory processes, Resources

## Abstract

**Hintergrund:**

Familienbildungsangebote unterstützen nicht nur familiale Interessen, sondern dienen auch der Gesundheitsförderung. Aktuelle Elternforschungen zeigen, dass Eltern durch das breite an sie gerichtete Anforderungsspektrum unter Druck geraten und sich belastet fühlen, was wiederum Auswirkungen auf das gesamte familiale Wohlbefinden haben kann. Um stressassoziierten Erkrankungen im Zusammenhang mit Überforderungserleben entgegenzuwirken, brauchen Eltern, wie alle anderen Familienmitglieder auch, günstige Rahmenbedingungen und Ressourcen.

**Ziel:**

Im Rahmen eines Landesmodellprojekts Fachstelle für Familienbildung – ALFA (Alles ist Familie – Familie ist alles) in Mecklenburg-Vorpommern wurden Potenziale und Mechanismen präventiver Familienbildungsangebote untersucht, welche Eltern in ihren Kompetenzen als auch in der Förderung ihrer Gesundheit unterstützen können.

**Methode:**

In leitfadengestützten Gruppeninterviews wurden Teilnehmer:innen von Familienbildungsangeboten im Übergang zur Elternschaft befragt. Die Datenanalyse und Theoriebildung erfolgte nach der Grounded-theory-Methodik, um so Wirkungszusammenhänge tiefergehender zu verstehen.

**Ergebnisse:**

Familienbildungsangebote stärken Er- und Beziehungskompetenzen auf Eltern-Kind-Ebene und fördern ein breites Spektrum an psychischen und sozialen Ressourcen. Familienbildung wirkt präventiv und fördert darüber hinaus salutogenetische Dynamiken durch Anregung psychosozialer Regulationsprozesse. Die von den Nutzer:innen erlernten Fähigkeiten und psychosozialen Ressourcen sind sowohl für die Herstellung eines gelingenden Familienalltages sowie zur Förderung individueller und familialer Gesundheit relevant.

**Schlussfolgerung:**

Die empirischen Ergebnisse unterstreichen, dass es sinnvoll ist, Angebote zur Familienbildung und psychosozialer Gesundheitsförderung trägerübergreifend zu gestalten, da beide Bereiche sich auf die Förderung elterlicher Ressourcen und Gesundheitskompetenzen beziehen. Dabei sind Wirkweisen, Schnittmengen sowie Abgrenzungen in der Gestaltung gesundheits- und familienbezogener Präventionsangebote tiefergehend zu untersuchen.

## Abstract (en)

**Background:**

Parenthood is a responsibility that is associated with numerous requirements and competencies in addition to care and upbringing skills. Current parental research shows that parents feel increasingly under pressure and burdened, which in turn can have an impact on overall family well-being. To counteract stress-associated illnesses in connection with excessive demands, parents—like all other family members—need favorable conditions and resources.

**Aim:**

In project ALFA (“Alles ist Familie – Familie ist alles”), the potential and mechanisms of preventive family support offers which can support parents in their competences and in promoting their psychosocial resources were examined.

**Methods:**

In guideline-based group interviews, participants in family support offers were interviewed during the transition to parenthood. Data analysis and theory formation were carried out according to the grounded theory methodology to better understand interdependencies.

**Results:**

Family support offers strengthening parenting and relationship skills at parent–child level and promotes a wide range of psychological and social resources that are relevant for the functioning of everyday family life as well as for promoting individual and family health. Family support catalyzes salutogenetic dynamics by stimulating psychosocial regulatory processes in the context of parenthood. The skills and psychosocial resources learned by the users are relevant both for creating a successful everyday family life and for promoting individual and family health.

**Conclusions:**

Acquired psychosocial resources can support parents in parenting and increase health competency, whereby the two areas are associated with each other in a transactional manner. Thus, offers combining support for families and health promotion appear advantageous and need to be more investigated in greater depth.

## Einleitung

Familien sind durch mehrgenerationale Beziehungen geprägt, in denen eine wechselseitige informelle Sorge um das körperliche, emotionale und geistige Wohl im Zentrum steht [56]. In diesen Sorgebeziehungen werden Eltern mit vielfältigen Familienentwicklungsaufgaben, je nach soziokulturellem Hintergrund, verantwortet [48]. Elternanforderungen sind folglich nicht etwa „qua Natur und Menschheitsgeschichte“ vorbestimmt oder allein an biologische Elternschaft geknüpft, sondern „in ihrer sozialen Konstruktion abhängig von der gesellschaftlichen Definition des Kindes, von pädagogischen Leitbildern und Maßstäben, die sich im Lauf der Jahrhunderte ändern“ [9]. Die Förderung und Aufrechterhaltung des familialen Wohlbefindens muss aktiv hergestellt werden, weil ihr Entstehen und ihre Kontinuität sich nicht selbstverständlich und fraglos ergibt [32]. Familie ist demzufolge, weniger als eine gegebene Ressource zu verstehen, sondern erfordert insbesondere von Eltern die stetige Herstellung gemeinsamer Zeiträume sowie die Erbringung von Sorgearbeit, was Jurczyk unter dem praxeologischen Konzept des „doing family“ konkretisiert [33]. Familiale Sorgearbeit wiederum subsumiert eine Vielfalt an Tätigkeiten, wie betreuen, erziehen, fördern, zuwenden, pflegen, organisieren und versorgen. Im Fokus von Wissenschaft und Praxis steht oftmals die elterliche Sorge in Bezug zum Kind. Inwieweit Eltern für ihr Wohlbefinden selbst sorgen, scheint gesellschaftlich weniger relevant [17, 33], obgleich Selbstsorge wichtig ist, um für andere Sorgen zu können.

Neben dem Recht und der Pflicht von Eltern, vielfältige Sorgeaufgaben für ihre Kinder zu erfüllen, steigen im Kontext gesellschaftlicher Wandlungsprozesse weitergehende Anrufungen an Mütter und Väter, im Sinne von diskursiven Anforderungen darüber, wie Elternsubjekte sein sollen [16, 19]. Gelebte Elternschaft steht hierbei häufig im Spannungsfeld unterschiedlicher gesellschaftlicher Erwartungshaltungen [48]. Dabei sind Eltern nicht allein für die Sorge verantwortlich, sondern werden vermehrt zur Förderung von Bildungschancen aufgefordert, wobei Familie verstärkt als Bildungsort funktionalisiert wird, in dem Kinder von Beginn an – möglichst schon im Mutterleib – zu fördern sind [33]. Dies kann jedoch auch zu steigendem Bildungsdruck nicht nur für Kinder, sondern auch für Eltern führen [33]. Doch nicht nur der Bildungsdruck, sondern auch der Zeitdruck, der finanzieller Druck und der normativ aufgeladene Erziehungsdruck in Verbindung mit einem Anspruch an kompetenter und verantworteter Elternschaft können mit gesundheitlichen Belastungen und eingeschränktem Wohlbefinden der Eltern korrelieren [12, 20, 21]. Über das subjektive Elternerleben bleibt die Anrufung an verantworteter Elternschaft jedoch konsistent: „Kinder zu haben, so die Botschaft an Frauen und Männer, ist eine überaus verantwortungsvolle Aufgabe, die mit hohen Anforderungen an die eigenen Fähigkeiten und Ressourcen für eine optimal ablaufende Elternschaft verknüpft ist“ [34, S. 1].

Dass Bildungs- und Sorgeaufgaben (Care-Arbeit) durch Eltern neben zu erfüllender Erwerbsarbeit systemrelevant sind, zeigte sich sehr deutlich im Zuge der COVID-19-Pandemie („coronavirus disease 2019“). Eltern thematisierten in der Pandemieerfahrung zunehmend kräftezehrende Belastungen und eine unzureichende Unterstützung [1, 30, 58, 59]. Auf der anderen Seite wurde es Eltern in dieser Zeit jedoch auch möglich, ihren „normalen“ Familienalltag *vor* der Coronaphase kritisch zu hinterfragen. So machten sich Eltern in der Pandemie bestehende familiale Stressoren bewusst, wie Freizeit- und Beziehungsstress oder ungleich verteilte Sorgearbeit, aber es wurde Eltern auch deutlich, wie wichtig und erfüllend familiale Beziehungen für das Wohlbefinden sein können [1, 60]. Zudem wurde deutlich, dass bereits vor pandemischen Ausnahmezuständen die innerfamiliale Aushandlung und Vereinbarkeit von Erwerbsarbeit und Familienarbeit ein oftmals stressbesetztes Dauerthema für Eltern ist, welches in einer Selbstverständlichkeit unsichtbar bleibt, bis eine Krise kommt, die wie das vielzitierte Brennglas wirkt [27]. Doch wenn es um den Wert von Arbeit geht, steht Erwerbsarbeit immer noch stets im Zentrum des sozialstaatlichen Fokus, während Care-Arbeit immer noch unzureichend mitgedacht und anerkannt wird [17]. Diese herabsetzende Positionierung steht jedoch der Ansicht entgegen, dass Familienarbeit als Elternaufgabe nicht allein eine private Angelegenheit darstellt, sondern von öffentlichem Interesse ist, weil die Form der Arbeit als eine gesamtgesellschaftlich unverzichtbare Leistung für das Gelingen von Gesellschaft gilt [17, 33].

Doch wie erleben Eltern sich in ihren Familienaufgaben und gesellschaftlichen Anrufungen in Bezug auf ihr eigenes Wohlbefinden. Das ständige Ausbalancieren zwischen Kindererziehung, Familienarbeit und Beruf kann bei Eltern zu „Energie- und Aufmerksamkeitskonkurrenzen“ führen [33, S. 157]. Eltern können in der Folge vermehrt Stress wahrnehmen und müssen Prioritäten setzen, wobei Studien belegen, dass Eltern weniger die Zeit für Kinder reduzieren, sondern vielmehr die ihrer Partnerschaft und für die eigene Regeneration zurückstellen [50]. Die Wahrnehmung von Belastungen kann „Eltern unter Druck“ [28] setzten und zu „erschöpften Familien“ [37] führen, was sich auch auf die Gesundheit von Familienmitgliedern und auf das Familienklima niederschlagen kann [20, 38]. In risikoreichen und ressourcenarmen Belastungskonstellation bilanziert Jurczyk [33], dass Eltern gerade noch das pragmatische Vereinbarkeitsmanagement gelingen, aber für die Herstellung von Wir-Gefühl und Zusammenhalt, dem förderlichen „doing family“, fehlt häufig die Kraft. Ziel dieses Beitrags ist es, Potenziale und Mechanismen präventiver Familienbildungsangebote aus der Empirie aufzuzeigen, die Eltern in ihren Kompetenzen als auch in der Förderung ihres Wohlbefindens unterstützen können. Nach der Ergebnisdarstellung folgen Überlegungen zur trägerübergreifenden Gestaltung von Angeboten der Familienbildung und psychosozialen Gesundheitsförderung, da beide Bereiche sich auf die Förderung elterlicher Ressourcen und Gesundheitskompetenzen beziehen und integrativ weiter zu entwickeln sind.

## Hintergrund

Unter dem Begriff der Familienbildung wird i. Allg. ein eigenständiger, mit anderen Arbeitsfeldern der Kinder- und Jugendhilfe verbundener Bereich verstanden, der für Familien in allen Lebensphasen und -lagen zuständig ist. Eltern sollen durch diese Angebote Unterstützung und Anregungen für einen gelingenden Familienalltag erhalten und zwar unter Berücksichtigung ihrer vielfältigen Bedürfnisse, Ressourcen, Erfahrungen, Fähigkeiten sowie Lern- und Handlungsgründen [36].

Als kommunales Präventionsangebot finden Familienbildungsmaßnahmen ihre rechtliche Fundierung v. a. im § 16 des SGB VIII als allgemeine Familienförderung. In Kontext eines präventiven Ansatzes sollen Leistungen nach § 16 SGB VIII „Erziehungsberechtigte bei der Wahrnehmung ihrer Erziehungsverantwortung unterstützen und dazu beitragen, dass Familien sich die für ihre jeweilige Erziehungs- und Familiensituation erforderlichen Kenntnisse und Fähigkeiten insbesondere in Fragen von Erziehung, Beziehung und Konfliktbewältigung, von Gesundheit, Bildung, Medienkompetenz, Hauswirtschaft sowie der Vereinbarkeit von Familie und Erwerbstätigkeit aneignen können und in ihren Fähigkeiten zur aktiven Teilhabe und Partizipation gestärkt werden. Sie sollen auch Wege aufzeigen, wie Konfliktsituationen in der Familie gewaltfrei gelöst werden können.“ Ein weiterer rechtlicher Bezugspunkt sind die Erwachsenen- und Weiterbildungsgesetze der Länder. Sie heben stärker den Anteil der Erwachsenenbildung als Aspekt des lebenslangen Lernens im Kontext der Familienbildung hervor.

Familienbildung möchte erreichen, dass das Zusammenleben innerhalb der Familie gelingt, dass die gesellschaftliche Teilhabe und Ressourcen zur Gestaltung des Familienalltags gestärkt werden und dass Möglichkeiten der Orientierung für eine selbstbestimmte familiale Lebensführung geboten werden [46, S. 61 f.]. Im Bereich Familienbildung ist Gesundheitsförderung und -vorsorge eine wichtige Aufgabe, indem die Stärkung von Ressourcen und Kompetenzen als elementar und relevant angesehen wird. Die Ressourcen sind wiederum wichtig zur Herstellung des psychischen, sozialen und körperlichen Wohlbefindens im Rahmen von Familie. Folglich liefern Familienbildungsangebote sowohl in der Förderung von Gesundheit (im Sinne des körperlichen und psychosozialen Wohlbefindens) als auch in der Vorbeugung von psychischen und sozialen Risiken einen Beitrag und können so die Vermeidung von Erziehungs- und Beziehungsproblemen sowie von Kindeswohlgefährdung unterstützen. In diesem Verständnis zeigt sich eine Schnittstelle zwischen Familien- und Gesundheitsförderung.

Damit Eltern die komplexen Anforderungen in ihrem Familienleben gelingend bewältigen können, stellt die Förderung ihrer Gesundheit eine wichtige Schlüsselfunktion dar. Gesundheit in Bezug auf Elternschaftspraxen kann mit Becker [8, S. 188] begriffen werden als „die Fähigkeit zur Bewältigung externer und interner (psychischer) Anforderungen“. Gesundheit ist demnach verbunden mit dem Streben nach einem Balancezustand zwischen Gesundheitsbelastungen und Gesundheitsressourcen. Folglich ist Gesundheit also ein „nicht selbstverständliches Gleichgewichtsstadium von Risiko- und Schutzfaktoren, das zu jedem lebensgeschichtlichen Zeitpunkt erneut hergestellt werden muss“ [8, S. 188]. Damit wird deutlich, dass nicht nur Familie im Sinne des „Doing-familiy“-Konzepts sozial hergestellt werden muss, sondern auch Gesundheit im Sinne eines „Doing-health“-Konzepts von besonderer Bedeutung ist.

In einem ganzheitlichen Verständnis werden Familien also nicht nur durch Familienförderung, sondern auch durch Gesundheitsförderung unterstützt. Einen zentralen Stellenwert im Gesundheitsbereich erhält Familie, indem die World Health Organisation (WHO) einen Zusammenhang in der Ottawa Charta eindeutig konstatiert: „Gesundheit wird von Menschen in ihrer alltäglichen Umwelt geschaffen und gelebt: dort, wo sie spielen, lernen, arbeiten und lieben“ [44]. Der Schlüssel für die Förderung der Gesundheit der Menschen liegt demnach in ihren Lebenswelten, zu denen auch Familienwelten gehören. Und nach Thiessen [55, S. 356] ist es notwendig, „Familien in ihrem Eigensinn als wesentliche Orte für die Gewährleistung und aktive Mitgestaltung gesundheitsförderlicher Lebensbedingungen und Kompetenzen in den Blick zu nehmen“. Aus sozialrechtlicher Perspektive ist die Stärkung von Eltern und Kindern folglich nicht nur nach § 16 SGB VIII relevant, sondern auch in den Leistungen der gesetzlichen Krankenversicherungen nach § 20 und § 20a SGB V betont. Familienbezogene Gesundheitsförderung ist insofern ein präventives Handlungsfeld, das im Präventionsleitfaden nach § 20 Abs. 2 SGB V geregelt ist. Demnach sollen trägerübergreifende Angebote der Prävention und Gesundheitsförderung an Familien mit „besonderen Bedarf an Unterstützung“ gefördert werden [35, S. 40]. Adessat:innen sind werdende und junge Familien, Alleinerziehende und schwer erreichbare, sozial benachteiligte Familien, wobei die Angebote für Familien möglichst niedrigschwellig in Einrichtungen in der Kommune (z. B. Familien‑, Bürgerzentren, Stadtteiltreffs) umgesetzt werden sollen [35, S. 39 f.]. Im Präventionsleitfaden wird auch festgestellt, dass über das gemeinsame Ziel der Stärkung der Gesundheitskompetenz Berührungspunkte zu den Leistungen nach § 16 SGB VIII bestehen [35, S. 153]. Kooperation mit beispielsweise Familienbildungszentren sowie Vereinen (z. B. für Migrantinnen und Migranten, Stadtteiltreffs) sind daher als möglich anzusehen. Der Präventionsleitfaden konstatiert außerdem, dass Krankenkassen sich im Setting Kommune anteilig an gesundheitsförderliche Inhalte beteiligen können. Gemeint sind hierbei evaluierte verhaltensorientierte Programme, die nicht explizit in den Zuständigkeitsbereich der gesetzlichen Krankenkassen fallen, die jedoch gesundheitsförderliche Aspekte berücksichtigen und entsprechende Effekte versprechen [35, S. 40]. Welche Programme damit gemeint sind, wird allerdings nicht weiter expliziert. Buschhorn [12] konstatiert, dass auf nationaler Ebene sowohl eine Modellierung und Erhebung von familienunterstützenden Programmen als auch eine Erfassung angebotsübergreifender Wirkfaktoren zur Überprüfung der Effekte, die mit unterschiedlich ausgestalteten Angeboten intendiert werden, bisher kaum erfolgt sind. Studien, welche anhand formulierter Wirkfaktoren übergreifende Effekte familienbegleitender und -unterstützender Angebote untersuchen, existieren bislang nur im internationalen Kontext [12].

## Forschungsstand zu Elternschaft und Gesundheit

In Elternforschungen wurden verschiedene Risikokonstellationen und Störungen untersucht, die insbesondere mit Blick auf die psychische Gesundheit von Kindern einen negativen Einfluss haben. Dabei wird die Bedeutung von niedriger sozialökonomischer und psychosozialer Ressourcenausstattung in Elternschaft als (dys)funktionale und riskante Variable in Bezug zum Kindeswohl betont [10, 15, 40, 45, 57]. So wurde in den verschiedenen Studien nachgewiesen, dass der Grad an gesundheitlichem Wohlbefinden und des Stresserlebens von Eltern einen Einfluss auf das Erziehungsverhalten und die kindliche Entwicklung haben kann. Auch zeigt ein systematischer Review von Santvoort et al. [47], dass Kinder von Eltern mit Angststörungen ein erhöhtes Risiko haben, ebenfalls eine Angststörung zu entwickeln und Kinder depressiv erkrankter Eltern können ein breites Spektrum psychischer Störungen entwickeln. Bolster et al. [11] resümieren, dass die Reduzierung elterlicher Belastungen einen wichtigen Ansatzpunkt darstellen kann, um die psychische Gesundheit von Kindern und Jugendlichen zu verbessern.

Wie oben schon erwähnt, lässt sich Gesundheit von Kindern und Eltern nicht allein durch die Abwesenheit von Erkrankungen bzw. Störungen erklären, sondern es geht auch darum, in einem salutogenetischen Blick festzustellen, inwieweit gesunde Anteile im Menschen kompetenzfördernd in Richtung Selbstregulationsfähigkeiten aktiviert werden können [2, 7]. Wissenschaft und Praxis betonen in den letzten Jahren immer wieder die Bedeutung der Ressourcenorientierung im Bildungsbereich Familie und Gesundheit [5, 23, 26]. Friedemann [22] konstatierte, dass Ressourcen im Kontext Familie, wie sichere Bindung und Beziehung, Problemlösefähigkeit, Selbstwirksamkeit und ein weitgehend stabiles Familienklima mehr in den Blick genommen werden sollten, „um zu einem umfassenden Bild und einer Abwägung zwischen Belastungen und Ressourcen gelangen zu können“. Hierbei sind nicht allein die Risiken und Ressourcen des Kindes, sondern der gesamten Familienmitglieder, also auch der Eltern, von Relevanz für das familiale Wohlbefinden [29]. Schnabel [49] führte in diesem Kontext das Modell der Familiensalutogenese aus, das sich auf Ressourcen und auf die Entstehung von Gesundheit im Familiensystem bezieht, um so die pathogenetische Perspektive auf Familie zu erweitern.

Dass Elternschaft sowohl mit gesundheitsrelevanten Risikofaktoren als auch mit Ressourcen in einem Zusammenhang steht, verdeutlicht sich in Familienmonitorings [3, 4]. So wurde in der Familienstudie von 2018 [3] konstatiert, dass etwa drei Viertel der befragten Eltern ihren eigenen Gesundheitszustand als sehr gut oder gut beurteilen und somit jedes vierte Elternteil die eigene Gesundheit als mittelmäßig bis schlecht einschätzt. Innerhalb der Stichprobe werden Unterschiede sichtbar, indem sich die Selbsteinschätzung der Gesundheit bei den befragten Eltern nach dem Bildungsabschluss, dem Geschlecht und der Elternkonstellation unterscheidet. So schätzen Eltern mit Abitur oder Hochschulabschluss ihren Gesundheitszustand durchschnittlich besser ein als Eltern mit Hauptschulabschluss (82 % als gut oder sehr gut im Vergleich zu 69 %) und weniger Mütter als Väter schätzen ihren Gesundheitszustand als gut bis sehr gut ein (ca. 82 % im Vergleich zu 72 %) und Elternpaare schätzen ebenso ihren Gesundheitszustand besser ein als alleinerziehende Eltern (77 % im Vergleich zu 69 %). Insgesamt lassen sich aus den Ergebnissen ableiten, dass Stresserleben und reduziertes Wohlbefinden für einige Elterngruppen größer ist als für andere Eltern, die mehrfachen Benachteiligungsrisiken ausgesetzt sind, weil sie z. B. keinen oder einen geringen Bildungsabschluss besitzen, in herausfordernden Familiensituationen leben oder weil sie weniger Ressourcen, Unterstützungsmöglichkeiten und Teilhabechancen haben. Eine Unterstützungsmöglichkeit für Eltern stellen Familienbildungsangebote dar, welche die Gesundheit und das Wohlbefinden aller Familien im Blick haben.

So kann anhand eines systematischen Reviews von Barlow et al. [6] verdeutlicht werden, inwiefern Familienbildungsangebote die psychosoziale Gesundheit von Eltern effektiv verbessern. Dazu wurden 48 randomisierte, kontrollierte Studien mit mindestens einer wissenschaftlich standardisierten Messgröße für die psychosoziale Gesundheit von Eltern in die Metaanalyse einbezogen. Überwiegend waren dies angloamerikanische Studien, aus Deutschland haben lediglich zwei Untersuchungen die Studienauswahlkriterien erfüllt. Diese beziehen sich auf das Triple P-Gruppenprogramm für Eltern und Kinder [18, 25]. In der Metastudie konnte ein kurzzeitiger Nutzen von Familienbildungsangeboten für Eltern im Hinblick auf Depression, Angstgefühle, Stress, Wut, Schuldgefühle, Selbstvertrauen und die Zufriedenheit in der Partnerbeziehung nachgewiesen werden. In dieser Metastudie wurde also eine Vielzahl an Outcome-Variablen untersucht, um psychosoziale Gesundheitsaspekte in präventiven Elternprogrammen zu bewerten. Eine Modellierung von Wirkfaktoren, die die Adressat:innenperspektive berücksichtigen, ist bisher jedoch noch nicht in präventiven Familienförderbereichen erfolgt [13].

In der Metastudie von Barlow et al. [6], als auch in weiteren Studien wurde außerdem konstatiert, dass es Forschungsarbeiten bedarf, welche die Mechanismen und förderlichen Rahmenbedingungen in Elternprogrammen identifizieren, die zur Entwicklung elterlicher Ressourcen und zur Förderung elterlichen Wohlbefindens beitragen [13, 42, 43]. Thiessen [55, S. 356] betont hierbei die Bedeutung der Verbindung zwischen „doing family“ und „doing health“, welche künftig systematischer zu erfassen sei. Mit Blick auf Wirkforschung erhalten qualitative Methoden eine bedeutsame Rolle, wenn es darum geht, Mechanismen sozialer Interaktion und gesundheits- sowie familienförderlicher Kontexte angemessen verstehen, beschreiben und analysieren zu können [13].

Die folgende empirische Untersuchung fand von 2015 bis 2018 im Rahmen eines Landesmodellprojekt „Fachstelle ALFA für Familienbildung“ (Alles Familie – Familie ist alles) statt, um Impulse für die fachliche Weiterentwicklung und Vernetzung bestehender Maßnahmen der Familienbildung in Mecklenburg-Vorpommern zu geben [41]. Dabei wurde der Frage nachgegangen, wie Familienbildungsangebote auf Eltern wirken und welche Wirkmechanismen sowie präventiven und gesundheitsförderlichen Aspekte sich aus Adressat:innenperspektive ableiten lassen.

## Methode

Zur Beantwortung der Fragen wurde ein qualitatives Forschungsdesign gewählt. Dies ermöglichte eine systematische und tiefgründige Analyse empirischer Daten und eröffnete damit Zugänge zum Verständnis von Familienbildung in sozialen Wirklichkeiten. In dieser qualitativ-empirischen Untersuchung wurden leitfadengestützte Gruppeninterviews mit Nutzer:innen durchgeführt, die Familienbildungsangebote im Übergang zur Elternschaft in Anspruch genommen haben. Zur Darstellung der Nutzer:innenperspektiven wurden insgesamt 5 Fokusgruppen mit je 5–7 Teilnehmer:innen institutioneller Familienbildungsangebote interviewt. Die Gruppeninterviews wurden hierbei im Rahmen von PEKiP®-Kursen in Familienbildungsstätten durchgeführt. PEKiP® (PEKiP e.V., Wuppertal, Deutschland) steht als markenrechtlich geschütztes Kürzel für „Prager-Eltern-Kind-Programm“. Dabei handelt es sich um ein Angebot zur Begleitung von Babys und deren Eltern durch die ersten 12 Monate. Das Programm wurde in Form von Präsenzkursen angeboten, wobei die einzelnen Kursstunden von zertifizierten Kursleiter:innen geleitet werden. Eine Kurseinheit dauert 90 min und findet einmal pro Woche statt. Die Interviewpersonen waren Mütter, Pflegemütter und ein Vater, die zum Zeitpunkt der Interviews bereits einige Kurseinheiten absolviert hatten, sodass die Teilnehmer:innen sich und das Angebot bereits kannten. Die Kursleiter:innen waren während des Interviews nicht im Raum. Das Datenmaterial wurde anschließend transkribiert und mithilfe eines computergestützten Verfahrens (f4 und MAXQDA) aufbereitet und für Analysen zugänglich gemacht.

Ausgehend von den unterschiedlichen Nutzer:innenperspektiven, orientierte sich die Datenanalyse und die Theoriebildung an der Grounded-theory-Methodik nach Strauss und Corbin [54], um an Mechanismen des sozialen Miteinanders im Familienbildungsbereich zu gelangen und um diese in einem handlungstheoretischen Bedingungsgefüge darzustellen. Semantische Bestandteile des Datenmaterials wurden in sinnvolle Analyseeinheiten segmentiert und offen kodiert. Es wurde in diesem ersten Analyseschritt nach sozialen Phänomenen im Datenmaterial gesucht. Phänomene, die sich in Textstellen zeigten, wurden als Indikatoren für ein darunterliegendes Konzept kodiert. Der zweite Schritt war das axiale Kodieren, in dem die Kodes zu abstrakten Kategorien verdichtet und reduziert wurden, wobei die Beziehungen zwischen den Kategorien in den Mittelpunkt der Analyse standen. Ferner wurde das Datenmaterial im selektiven Kodierschritt nach ähnlichen oder kontrastierenden Subkategorien analysiert. Im Rahmen fortschreitender Kodierarbeiten und permanenten Vergleichen verdichteten sich die Konzepte und trugen zur Entwicklung von Kategorien bei. So bildete sich im Kodierprozess eine Schlüsselkategorie heraus, die wiederum in definierten Beziehungen zu den anderen herausgearbeiteten Kategorien steht [39]. Für die Fragestellung, den Analyseprozess und das Kategorien-Beziehungs-Geflecht erwies sich das handlungstheoretische Kodierparadigma nach Strauss und Corbin (1996) als angebracht. Angeordnet wurden die entstandenen Kategorien im Kodierparadigma um eine Kernkategorie herum, welche den analytischen Leitgedanken verdeutlicht. Folglich wurde eine datenverankerte Theorie mittlerer Reichweite für den Bereich Familienbildung als konkreter Ausschnitt sozialer Wirklichkeit entwickelt [53]. Zur intersubjektiven Nachvollziehbarkeit als auch zur Qualitätsentwicklung und -sicherung fanden regelmäßig begleitende Besprechungen in Forschungswerkstätten statt.

## Ergebnisse

### Psychosoziale Regulation als Mechanismus in der Familienbildung

**Abb. 1:**
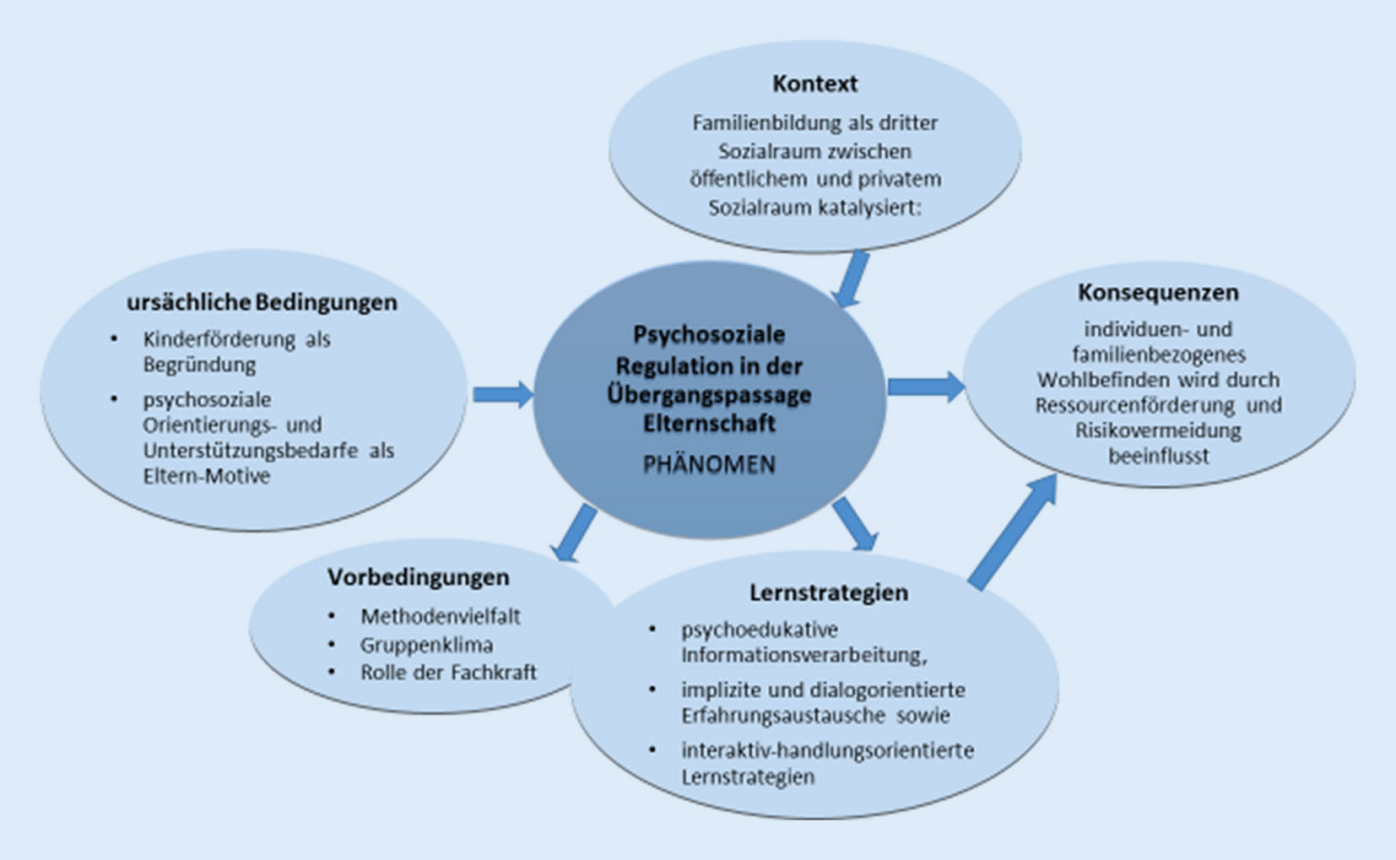
Psychosoziale Regulation als Mechanismus in der Familienbildung. (Quelle: eigene Darstellung)

Im Mittelpunkt des handlungstheoretischen Verständnisses von Familienbildung konnten aus den Elterninterviews psychosoziale Regulationsprozesse als zentrales Phänomen bestimmt werden, die sowohl für das individuelle als auch familiale Wohlbefinden von Relevanz rekonstruiert werden konnten. Dieser komplexe Prozess subsumiert vielfältige familienbezogene Gestaltungs‑, Anpassungs- und Bewältigungsanforderungen aus Elternperspektive, welche für die Interviewten in der Übergangspassage zur Elternschaft von Relevanz sind [41]. In Abb. 1 wird das zentrale Phänomen im handlungstheoretischen Bedingungsgefüge vorgestellt und in einer Gesamtstruktur der sich darauf beziehenden Kategorien entfaltet. In der Theoriedarstellung werden Interviewaussagen der Befragten *(B)* als Ankerzitate eingefügt, um die Abstraktionen exemplarisch und nah an der Empirie zu verdeutlichen.

### Ursächliche Bedingungen für das Phänomen

Der Mechanismus der psychosozialen Regulation wird eingeleitet durch *ursächliche Bedingungen*. Hier stellt der Übergang in Elternschaft aus Nutzer:innensicht ein prägnantes Lebensereignis dar, in dem die Übernahme der Elternrolle und damit verbundene Veränderungsprozesse zu regulieren sind. Der Übergang in Elternschaft ist für die Befragten ein relevanter Beweggrund für die Inanspruchnahme von Angeboten der Familienbildung. Als vordergründigen Inanspruchnahme – Sachgrund – nannten die Nutzer:innen von Familienbildungsangeboten den Wunsch, ihrem Kind von Anfang an bestmögliche Fördermöglichkeiten anzubieten „B: Im Mittelpunkt stehen die Kinder, dass die gefördert werden.“

Hinter diesem Sachgrund lassen sich jedoch verschiedene Motive der Inanspruchnahme auf Ebene des psychosozialen Wohlbefindens von Eltern erkennen. Insbesondere ein subjektiv wahrgenommener Mangel an familienbezogenen sozialen Strukturen und Systemen im sozialen Nahraum als auch bereits positiv gemachte Erfahrungen mit Familienbildung determinieren für Eltern implizit die Inanspruchnahme von Familienbildungsangeboten in ihrem sozialen Umfeld. „B: Weil eben keine Großfamilienstrukturen da sind. Oder auch so Dorfstruktur eigentlich nicht da ist. Wo man dann nebenan eine erfahrene Frau oder Freundin hat irgendwie. Und das muss dann so ein Familienzentrum auffangen*.*“

Neben dem Bedürfnis nach sozialem Anschluss und Verbindung mit anderen Personen in ähnlicher Lebensphase konstatieren Nutzer:innen ferner Empfindungen der Unsicherheit und Anspannung sowie Bedürfnisse nach Orientierung in Bezug auf ihr Elternwerden und -sein. Exemplarisch verdeutlicht sich der Übergang in Elternschaft als psychosoziale und körperliche Regulationsleistung und der Bedarf an Unterstützung in folgender Aussage: „B … so empfand ich es als unsicher, gerade so wo sie so klein war, wie machst du das alles am besten mit ihr. Das war, glaube ich, so die größte Herausforderung in den ersten Tagen und Wochen. Mit dem Schlafentzug klarzukommen und dann auch das so zu versorgen (…) Stillen. Klar, man hat die Hebamme, die hat einem viel gezeigt, aber wie gesagt, sie ist nur eine halbe Stunde am Tag da. Und die andere Zeit ist der Partner arbeiten und du bist ganz alleine. Wenn man dann die Großeltern nicht zwischendurch hat, um tatsächlich mal eine halbe Stunde für sich zu haben, ist man 24 h ja nur für das Kind da und hat kaum noch Zeit*.*“

Mit Familienbildung verbinden die Interviewten viele Anregungen im Umgang mit dem Kind und dass sich hier ein Raum für Entlastung und elternbezogener Regeneration anbietet. Auch die Möglichkeit der Freizeitgestaltung im Familienzentrum wurde betont, was ebenfalls zur Inanspruchnahme weiterer Familienbildungsangebote motivieren kann. Niederschwellige Formate bieten insofern einen ersten Zugang an und ermöglichen Verweisungswissen über familien- und gesundheitsbezogene Unterstützungsmaßnahmen entlang bestehender „Präventionsketten“ [14] in den Kommunen. Ferner stellen genutzte Familienbildungsangebote einen Raum für Begleit- und Orientierungshilfen dar: „B: Ich finde das schon gut, wenn das auch so was ist, wo man auch mal bisschen an die Hand genommen wird*.*“

In diesem Zusammenhang verdeutlichten die Befragten Unsicherheiten in der Übergangspassage, auch aufgrund einer Überflutung an medialen Informationen zur Gestaltung der Elternrolle und in der Frage, wie Sorgeaufgaben erbracht werden können.

Dass Familienbildung für Eltern in der Moderne zunehmend relevant ist, begründet Schmidt-Wenzel [48, S. 17] damit, dass Wissen, Stabilität und Orientierung an Eltern weniger transgenerational weitergegeben werden, was insbesondere durch gesellschaftliche Individualisierungstendenzen und durch das Wegbrechen von Mehrgenerationenfamilien begründet wird. Sie konstatiert: Was früher ein Mindestmaß an Orientierung ermöglichte und weitgehend selbstverständlich geschah, erfordert jetzt „um des gezielten Erfolges willen, behutsame Einführung und bewusste Aufmerksamkeit der Erziehungsperson“ [48, S. 61 ff.].

### Vorbedingungen und Lernstrategien

Damit die psychosozialen Regulationsprozesse in der Übergangspassage gefördert werden können, sind verschiedene einflussnehmende *Vorbedingungen* von Relevanz. So wird durch die Kombination von unterschiedlichen Wissensbeständen und vielfältigen Methoden in Kombination mit einer flexiblen und vertrauensvollen Fachkraft ein wichtiger Rahmen gegeben, wenn es darum geht, den Übergang zur Elternschaft zu bewältigen. Den befragten Eltern ist die Möglichkeit situativ und selbstbestimmt auf Anliegen eingehen zu können von Bedeutung für ihren eigenen Lernprozess. „B: Probleme ergeben sich ja einfach, dass man die hier so bespricht. Also eigentlich ist das hier ein Austausch, findest du in keinem Internet und in keinem Buch so wieder. Und das bleibt dann so hängen, finde ich*.*“ In den Interviews zeigte sich, dass familiale Übergänge nicht allein für Kinder, sondern auch für Erwachsene herausfordernd und komplex sind. In der Rekonstruktion der Elternperspektive wurde Folgendes ersichtlich: Psychische und sozialen Orientierungsbedarfe sowie emotionale und handlungsbezogene Unsicherheiten wollen reguliert werden, Elternkompetenzen sind zu entwickeln und die Anpassungsanforderungen in der Übernahme der Mutter‑, Vater‑, Elternrolle wollen mit dem Selbstbild in Einklang gebracht werden.

In der Bewältigung dieser Anforderungen bietet Familienbildung Methoden und Räume für verschiedenste *Lernstrategien* an. Zur Förderung psychosozialer Regulationsprozesse in familialen Übergängen sind psychoedukative Informationsverarbeitung, implizite und dialogorientierte Erfahrungsaustausche sowie interaktiv-handlungsorientierte Lernstrategien von Relevanz. Mit diesem Strategienmix wird zum einen der Transfer von Fachwissen gefördert: „B: Erziehungswissenschaftler, (…) die einfach mal erzählen, ok es gibt diese, diese, diese Möglichkeiten wie ihr Kind ohne anschreien usw. (…) sie zeigt einem dann auch, wie man das so gesund herstellen kann, (…) man kann das erst mal theoretisch mitnehmen und dann nachher so in die Tat umsetzen*.*“

Zum anderen wird Raum für implizites Erfahrungswissen gegeben, der für die befragten Nutzer:innen als besonders relevant erachtet wird: „B2: Man konnte sich austauschen, gerade mit den anderen Müttern. Ich sage mal, die anderen hatten alle schon mehr Erfahrung, weil die alle schon das zweite Kind oder das dritte Kind dann hatten. Und ich war eigentlich die Einzige mit dem ersten Kind*.*“ Eltern nutzen also im öffentlich definierten Rahmen von Familienbildungsangeboten niederschwellige Beratungs- sowie reine Informationsvermittlungsangebote von Expert:innen unterschiedlichster Bereiche und organisieren sich in diesem Raum auch ein lebensweltorientiertes, psychosozial unterstützendes Lernmilieu, in dem die Fachkraft tendenziell eine moderierende Funktion innehat.

### Kontext psychosozialer Regulation

Familienbildung bietet aus Elternperspektive vielfältige Wissensbestände, Lerngelegenheiten und Methoden außerhalb ihres privaten Lebensbereichs und lässt sich als dritten Sozialraum verstehen. Familienbildung ist also als ein *Kontext*, der zwischen den Sozialräumen des privaten Familienraums (mit dem Ziel der Eigeninteressenverfolgung) und des öffentlichen Raums (i. S. d. professionellen Unterstützung) entsteht. Dieser dritte Sozialraum ist gekennzeichnet durch eine mittlere Distanz der Eltern untereinander und mittelgroßem Engagement füreinander, wobei Fachkräfte methodisch unterschiedlich auf die psychosozialen Regulationsbedarfe eingehen kann [41]. In diesem Raum interagieren folglich nicht allein professionelle Fachkräfte mit Familie, sondern Eltern nutzen auch die Möglichkeit voneinander zu lernen. Unter diesen Kontextbedingungen entwickeln sich Prozesse der psychosozialen Regulation integrativ durch Verschränkung verschiedener Wissensbestände und in gegenseitiger Unterstützung und Förderung. Somit verdeutlicht sich, dass Eltern in ihrer psychosozialen Regulation nicht nur Unterstützungs- und Hilfebedarf über Expert:innenwissen in Anspruch nehmen wollen, sondern auch ein gewisses Bedürfnis nach „peer support“ in sich haben, in dem sie sich untereinander unterstützen, sei es emotional oder durch Austausch von Erfahrungswissen.

### Konsequenzen

In den Elterninterviews wird ersichtlich, dass der Mix aus formell und informell angelegten Familienbildungskontexten psychosoziale Regulationsprozesse katalysiert. In der *Konsequenz* schildern Eltern sich gefördert in ihren Kompetenzen, um Stress zu reduzieren und eine Ressourcen-Belastungs-Balance herzustellen. Es ist anzunehmen, dass diese Kompetenzen sowohl für das eigene Wohlbefinden als auch für einen gelingenden Familienalltag relevant sind. Um einen Eindruck zu erhalten, folgt ein Zusammenschnitt aus einem Gruppeninterview: „B6: Man kann sich austauschen über Probleme. (…) B3: Eben, wir quatschen ja nicht nur über die Kinder, sondern es geht ja auch immer um uns. Um die Partnerschaft. (…) B1: Völlig egal. Das ist ungezwungen und dann wird ein Thema in den Raum geworfen, wenn einen was bedrückt und der möchte darüber reden. (…) B4: Es ist einfach auch irgendwo was Entspannendes, finde ich. (…) B6: paar Kontakte knüpfen, mit Babys zusammen, mit Muttis zusammen, auch Zeit zum Ausruhen auch für die Mutti, weil die Kinder können dann hier bisschen spielen und ja alles ankrabbeln und machen und tun. (…) B4: … oder wenn ich selber auch genervt bin vom Job. Na ja und da sind die Erziehungswissenschaftler oder was weiß ich, so wie die Ernährungswissenschaftler, die einfach mal erzählen, ok es gibt diese, diese, diese Möglichkeiten wie ihr Kind ohne anschreien usw*.*“

Mit Blick darauf, welche Kompetenzen für Eltern relevant sind, zeigte sich, dass die befragten Eltern in dem Angebot verschiedene Problemlösefähigkeiten und Umgangsweisen im Zusammenhang mit familialen Konflikten und Stresssituationen lernten und ihre Erziehungsfähigkeiten stärken konnten. Neben diesen präventiven Aspekten wird auch deutlich, dass im Familienbildungsbereich salutogenetische Dynamiken gefördert werden, die zur Erweiterung von gesundheitsförderlichen Lebenskompetenzen sowie zur Persönlichkeits- und Familienentwicklung beitragen können [31]. Familienbildung bietet den Befragten zufolge zum einen sicherheitsstiftende Orientierungen in der Übergangspassage zur Elternschaft und trägt zur Bildung und Erweiterung von Lebenswissen und alltagsnahen Handlungsfähigkeit bei. Die Gestaltung positiver Eltern-Kind-Bindungserfahrungen wird ebenfalls durch die Interaktion unter familienfreundlichen sowie zeitlichen und räumlichen Bedingungen geschildert, sodass die Beziehung von Eltern und den Kindern untereinander in ihren Lebenswelten gefördert wird. Die Befragten erlebten sich in ihrer subjektiven Lebensqualität insgesamt zufriedener durch die Inanspruchnahme des Angebots. Bedeutsam ist dabei die Erfahrung an Entlastung und Entspannung sowie die Erweiterung sozialer Unterstützungsressourcen durch das Angebot. Und auch die Netzwerkbildung sowie -erweiterung, z. B. zu anderen Eltern, aber auch zu Fachkräften aus Institutionen wie Kita, Jugendamt und Gesundheitseinrichtungen, wurde aus Sicht der Befragten durch das Angebot begünstigt.

Familienbildungsangebote sind folglich nicht nur Familienförderung, sondern implizieren auch Gesundheitsförderung im Sinne eines dynamischen Strebens nach einem Balancezustand zwischen Gesundheitsbelastungen und Gesundheitsressourcen [8]. Umfassend verstanden ist Familienbildung ein Querschnittbereich für vielfältigen Orientierungs‑, Kompetenz- und Unterstützungsgelegenheiten in allen familialen Lebenslagen und -phasen. Der Bereich der Stärkung von Erziehungskompetenzen selbst ist dabei von familialem und gesundheitsbezogenem Interesse, wobei Erziehung vor dem Hintergrund gesellschaftlicher Anrufungen an Elternschaft normativ aufgeladen scheint und damit Stress auf Seiten von Eltern entstehen kann. So erklärte eine Nutzerin, dass Erziehung, gerade, wenn diese als problematisch erlebt wird, ein „B4: ganz sensibler Bereich“ sei, sodass bei der Vorstellung der Normalitätsabweichung in Elternschaft, Stigmatisierungsangst zu vermuten ist. Zudem konstatiert eine weitere Nutzerin ihre Befürchtung, negativ etikettiert zu werden: „B6: Ich möchte auch nicht dastehen als die absolut überforderte Mutter, sondern dass man einfach so ganz lockere Runde (…). Die ganz normalen Probleme, die man sonst auch hat*.*“ Die Thematisierung insbesondere von Be- und Erziehungskonflikten werden von Nutzer:innen folglich eher in Einzelgesprächen gesucht, als implizit Gruppensettings darauf Bezug zu nehmen. Zugleich bieten Gruppenangebote, die weniger problembezogen an der Stärkung von Elternkompetenzen ansetzen, Gelegenheiten für nebenbei laufende, intensivere Beratungsgespräche, ohne dass Nutzer:innen sich selbst zu sehr exponiert in evtl. normabweichende Problematiken adressiert erleben. Familienbildung bietet folglich flexible Möglichkeiten, Stress in Erziehungs- und Beziehungsbereichen in einem gruppenbezogenen oder vertraulich-situativen Ansatz bearbeiten zu können: „B3: Na ja, das ergibt sich immer so. Also hier in dieser Gruppe ist es auch so aus dem Moment heraus und das sind so Sachen, die dann nebenbei laufen.“ Neben Vorträgen und anderen Gruppenangeboten werden insofern auch situative Beratungsgespräche mit Fachkräften genutzt. Der Scheinwerfer auf die Methodenvielfalt und die Offenheit in der Adressierung aller Eltern wirkt insofern weniger stigmatisierend und kennzeichnet in der Familienbildung eine niederschwellige Zugangsmöglichkeit zu vielfältigen schwierigen Problemsituationen von Familien, die im Schatten bestehen können und mit einem professionellen Blick bearbeitbar werden, sofern Vertrauen aufgebaut wurde.

### Ergebniszusammenfassung

Die ursächlichen Bedingungen, Lernstrategien als auch die Vorbedingungen, welche vorgestellt wurden, richten sich auf das zentrale Phänomen in der Familienbildung, in dem es im Kern um psychosoziale Regulationsprozesse geht. Aus den Interviews bringt folgendes Zitat die Beschreibung des Kerngedankens von Familienbildung wie folgt auf den Punkt: „Um mal den Kreis zu schließen. Sind solche Kurse natürlich nicht schlecht.“ Dieses Zitat unterstreicht das Schließen von Kreisen der Nutzer:innen im Rahmen von Familienbildungsangeboten, wenn es um Anpassung und Orientierung in Elternschaft geht. Die Symbolik des Kreisschließens kann folglich bedeuten, dass Wissen und Energie sich verdichtet und eine Quelle bzw. Ressource darstellt. Kreise für sich zu schließen, impliziert hierbei die Fähigkeit zur Selbstbestimmung und kann mit der Erfahrung von Selbstwirksamkeit einhergehen, da Teilnehmer:innen sich entscheiden, was diese aus Familienbildungsangeboten integrieren wollen und damit auch festlegen, was nicht integriert werden soll, um so ein gelingendes Zusammenleben in ihrem ganz individuellen Familienalltag zu realisieren.

Die befragten Nutzer:innen befördern somit in selbstbestimmter Weise ihre psychosozialen Regulationsprozesse durch den Erwerb an Handlungs- und Bewältigungswissen in Bezug auf vielfältige Themen wie Gesundheit, Umgang mit dem Kind und eigenen Stressoren sowie Ressourcen. So bilanziert eine Befragte die Inanspruchnahme von Familienbildungsangeboten wie folgt: „…von der Sache, man kann das erst mal theoretisch mitnehmen und dann nachher so in die Tat umsetzen. Und das finde ich (…) sowas sollte es nicht nur für uns geben, sondern für Alle, dieses Angebot, aber ja*.*“

Familienbildung verfolgt insofern und über die Prävention familienrelevanter Risiken hinaus auch die Förderung und Stärkung von Elternkompetenzen und Selbstregulationsfähigkeiten, die für das individuelle als auch familiale Wohlbefinden bedeutsam sind. Familienbildung impliziert hierbei zwei komplementäre Strategien mit unterschiedlichen Wirkrichtungen, während die Präventionsstrategie auf die Vermeidung und Reduktion von Risikofaktoren abzielt, fokussiert die Förderstrategie die Stärkung von Schutzfaktoren und Ressourcen [31].

## Diskussion

Die Ergebnisse zeigen zusammenfassend auf, dass Elternschaft – hier am Beispiel der Übergangspassage zur Elternschaft – mit psychosozialen Regulationsprozessen einhergeht und durch Entwicklung und Aktivierung von Ressourcen auf sozialer als auch psychischer Ebene gekennzeichnet ist. Psychosoziale Regulation stellt ein Mechanismus im elterlichen Entwicklungs- und Anpassungsprozess dar, wobei verschiedenste Bedingungen im Kontext von Familienbildung als Prozesskatalysatoren fungieren können. Dabei geht es im Familienbildungsbereich nicht allein darum, Risiken zu vermeiden, sondern auch um Ressourcenaktivierung und -stärkung zu ermöglichen. Psychosoziale Ressourcen sind wiederum für eine individuelle als auch familiale Lebensgestaltung und -bewältigung wichtig [41]. Für Eltern können diese Ressourcen sowohl in der Herstellung von Familie – dem „doing family“ – bedeutsam sein, als auch in der Aufrechterhaltung und Förderung von Gesundheit und zwar in Bezug auf sich selbst *und* in Bezug auf das Familiensystem [49]. Insofern haben psychosoziale Ressourcen für Eltern eine Scharnierfunktion zwischen der Herstellung von Familie und der Förderung von Gesundheit inne, wobei sich die beiden Bereiche wiederum transaktional aufeinander beziehen können [24, 29, 49, 52].

### Elternunterstützung als Querschnittsaufgabe in kooperativen Partnerschaften

Ausgehend von dieser reziproken „Verflochtenheit von Gesundheit und Familie“ [24, S. 11], sind Elternunterstützungsangebote folglich als Querschnittsaufgabe zu verstehen, zu gestalten und auszustatten. Die Förderung von Familie ist nicht allein in der Familienbildung zentral, sondern auch ein relevanter Bestandteil in der Gesundheitsförderung und damit, laut Präventionsgesetz der Krankenkassen, trägerübergreifend zu gestalten. Der GKV-Spitzenverband konstatiert in diesem Zusammenhang, dass alle Arten von Arbeit, also nicht allein berufliche Arbeit, sondern auch Familienarbeit und die Pflege von Angehörigen in Verbindung mit chronischem Stress als gesundheitlicher Risikofaktor stehen können [35].

Um familienbezogene Gesundheitsprogramme oder gesundheitsbezogene Familienprogramme gelingend zu gestalten, bedarf es neben fundiertem Wissen um die Wirkmechanismen, Effekte und Rahmenbedingungen auch kooperative Partnerschaften zwischen den Bereichen der Gesundheitsförderung und Familienbildung, um – fernab von Ressortlogiken – das psychische, soziale und körperliche Wohlbefinden von Eltern und damit auch von Kindern umfassend aufrechterhalten und fördern zu können.

In dieser Studie wurde deutlich, dass sich Eltern als Adressat:innen von Präventionsangeboten eine Weiterentwicklung dialogorientierter kooperativer Strukturen mit familienrelevanten Akteuren und Einrichtungen in sozialen und gesundheitlichen Bereichen praktizierten, damit die sie ihre familienrelevanten Übergänge fließend, gelingend und unkompliziert gestalten können. Insbesondere wenn es um sog. schwierige Themen, Probleme und Herausforderungen geht, ist es für die befragten Eltern von Relevanz, niederschwellige Angebotsformate als Türöffner für rechtzeitige Unterstützung zu haben, wobei Fachkräfte in ihrer Funktion als hilfreich erlebt werden, wenn es Eltern darum geht, Wissen vermittelt zu bekommen und Schnittstellen zu weiteren Hilfeleistungen zu überwinden.

### Förderung von familialem Wohlbefinden in lebensweltsensiblen Sozialräumen

Wissensvermittlung und die Stärkung psychosozialer Ressourcen von Eltern sind dem verhaltensorientierten Ansatz der Prävention und Förderung von Gesundheit und Familie zuzuordnen. Zugleich sind den Befragten familienfreundliche Lebens- und Gestaltungsspielräume für ihr familiales und eigenes Wohlbefinden von Bedeutung. Die Schaffung eines dritten Sozialraums – zwischen privatem Familienraum und öffentlichen Räumen für Gesundheit und Soziales – spiegelt insofern einen lebensweltorientierten Ansatz wider. Jener familienfreundliche Sozialraum schafft verschiedenartige Lerngelegenheitsstrukturen, in welchem Verhaltensweisen sowie soziale und kulturelle Ressourcen, wie Beziehungsgestaltung oder Netzwerkbildung aus Sicht der Nutzerinnen gefördert werden. Durch den Kontakt in mittlerer Distanz im dritten Sozialraum können Schwellenängste zwischen Nutzer:innen und Fachkräften reduziert werden, was wiederum eine Brücke sein kann, um Schnittstellen zu weiteren gesundheitsrelevanten und sozialen Hilfeleistungen und Einrichtungen im Bedarfsfall zu überwinden.

Nutzer:innen wünschen sich insofern Unterstützungsangebote, die lebensweltorientiert und verhaltensbezogen an direkte Alltagserfahrungen der Befragten anknüpfen. Sie wollen in unterschiedlichsten sozialen und gesundheitsrelevanten Lebensbereichen und Lebenssituationen gesehen und gestärkt werden. Zudem ist es für die befragten Eltern von Relevanz, dass die Angebote eine begleitende und unterstützende Plattform in der Gestaltung, Bewältigung und Anpassung in inner- und außerfamilialen Übergängen bieten. Eltern wünschen sich zudem Fachkräfte, die als vertrauensvolle Multiplikatoren fungieren, was sich als ein bedeutsames Merkmal von Niederschwelligkeit in der Inanspruchnahme herausstellt. Gerade im Bereich der Erziehung und dem damit verbundenen Stresslevels von Eltern (was immer noch als ein Tabuthema erscheint und ein sensibler Bereich ist) kommt die Bedeutung einer vertrauensvollen Beziehungsgestaltung durch Fachkräfte zum Tragen. Um Verunsicherung und chronischen Stress von Eltern vorzubeugen bzw. entgegenzuwirken und um folglich die Bereitschaft zu fördern, sich mit Familienproblemen offen und konstruktiv auseinander zu setzten, werden sensible werteorientierte Reflexionsgespräche als relevant erachtet.

### Elternadressierung in universeller Zielgruppenorientierung

Die Stärkung psychischer und sozialer Ressourcen ist sowohl im Bereich der Gesundheitsförderung als auch in Bereich der Familienbildung ein relevantes Ziel. Allerdings sind Angebote im Bereich der Eltern- und Familienbildung als Leistungen nach dem § 16 SGB VIII festgeschrieben und – in der Finanzierung als ein eigenständiger Bereich im SGB VIII verortet. Trägerübergreifende Angebote zur Verschränkung von Gesundheits- und Familienförderung werden als eine Aufgabe von den gesetzlichen Krankenversicherungen nach § 20 und 20a SGB V über das Präventionsgesetz (PrävG) geregelt. Derzeit wird Elterngesundheit im Rahmen der gesetzlichen Krankenversicherungen aber nicht unmittelbar entlang ihrer gesundheitsrelevanten Bedarfe gefördert, sondern erst im Zusammenhang mit weiteren einschneidenden familienrelevanten Veränderungsprozessen und Stressoren bedeutsam, wie Trennungen oder Arbeitslosigkeit. Hier entsteht also ein Dilemma zwischen dem Anspruch, Familienbildung als universellen Ansatz zu etablieren und gleichzeitig besonders belastete Elterngruppen in den Blick zu nehmen, die von der klassischen Familienbildung so nicht erreicht werden. Dieses Dilemma kann zwar nicht aufgelöst werden, dennoch haben die Ergebnisse jedoch verdeutlicht, das allgemein Orientierungs- und Unterstützungsbedarfe in der Gestaltung, Bewältigung und Anpassung an Elternschaft bestehen und in Anbetracht steigender gesellschaftlicher Anforderungen an Eltern mit starkem Stress besetzt sein können, sodass wir uns an dieser Stelle dafür aussprechen, einen universellen Ansatz zu stärken und Elternangebote weiterhin konzeptionell und prinzipiell stärker ressourcenorientiert zu gestalten. Um individuelle Bedarfe von allen Eltern angemessen zu berücksichtigen, würde ein universeller Ansatz im Adressat:innenzugang zudem dazu beitragen, dass Familienarbeit neben Erwerbsarbeit mehr Anerkennung erhält. Weiterhin würde eine allgemeine Adressierung an Eltern – ohne auf ein Alleinerziehend‑, Getrennt- oder Arbeitslossein zu rekurrieren – weniger stigmatisierend wirken, weil Eltern sich (zumindest im öffentlichen Raum) tendenziell nicht einer Gruppe zuordnen lassen wollen, die mit Vulnerabilität, Benachteiligung oder Überforderung konnotiert wird. Diese indirekte Ansprache könnte vermutlich dem Präventionsdilemma entgegenwirken, weil so nicht nur Eltern mit geringerem Bedarf erreicht werden, sondern sich auch Eltern angesprochen fühlen, die besondere Bedarfe und Bedürfnisse haben, aber deswegen nicht etikettiert werden wollen.

### Limitationen

Die Ergebnisse sind unter Berücksichtigung einiger Einschränkungen zu beurteilen. Im Allgemeinen sind die Limitationen dieser Arbeit hauptsächlich auf das verwendete Sample zurückzuführen. Das Sample bezieht sich fast ausschließlich auf Mütter mit Kleinstkindern und ist im Sampling durch das Einbeziehen von weiteren Kriterien zu kontrastieren, beispielsweise um Väter- oder Kinderperspektiven. Vor dem Hintergrund des eingeschränkten Samples entspricht das theoretische Modell in den Konzeptualisierungen nicht dem Idealzustand der theoretischen Sättigung [53]. Um die Gütekriterien dennoch zu erfüllen, wurden die Ergebnisse mit verschiedenen Forscher:innenperspektiven intersubjektiv nachvollziehbar trianguliert und kommunikativ validiert [51]. Weitere Forschung kann mit den Ergebnissen dieser Arbeit in systematischer und theoriegeleiteter Weise stattfinden, um einerseits die Analyseergebnisse weiter zu generalisieren und andererseits, um die Konzepte weiter zu verdichten und auszudifferenzieren. Letztlich sind die Erkenntnisse über Elternschaft und der reziproken „Verflochtenheit von Gesundheit und Familie“ [24, S. 11] weiterzuentwickeln.

## Fazit für die Praxis

- Die Studie erweitert den Blick auf Eltern- und Familienbedarfe.
- Es wurden psychosoziale Ressourcen im familienbildnerischen Kontext sichtbar, die für Eltern in einer Scharnierfunktion zwischen der Herstellung von Familie und der Förderung von familialer und individueller Gesundheit von Bedeutung sind.
- Die Förderung von Familien und somit auch von Elternressourcen ist als Querschnittsaufgabe zu verstehen und als kooperative Partnerschaft zwischen den präventiven Strukturbereichen zu Gesundheit und Familie weiterzuentwickeln.
- Im Wissenstransfer zwischen Wissenschaft und Praxis sind dafür Wirkmechanismen, Effekte und Rahmenbedingungen tiefergehend zu untersuchen, um Erkenntnisse über adressatenorientierte Formate zu erweitern und trägerübergreifende Zusammenarbeit zwischen Familienbildung und Gesundheitsförderung fundiert zu gestalten, bevor das Kind (oder das Elter) in den Brunnen gefallen ist.
